# Cell cycle reactivation of cochlear progenitor cells in neonatal FUCCI mice by a GSK3 small molecule inhibitor

**DOI:** 10.1038/srep17886

**Published:** 2015-12-08

**Authors:** M. Roccio, S. Hahnewald, M. Perny, P. Senn

**Affiliations:** 1 Laboratory of Inner Ear Research, Department of Clinical Research, University of Bern and University Department of Otorhinolaryngology, Head & Neck Surgery, Inselspital, Bern, Switzerland; 2 Department of Otorhinolaryngology, Head & Neck Surgery University Hospital Geneva (HUG), Switzerland; 3 Laboratory of Neuroinfectiology, Institute of Infectious Diseases (IFIK), University of Bern.

## Abstract

Due to the lack of regenerative capacity of the mammalian auditory epithelium, sensory hair cell loss results in permanent hearing deficit. Nevertheless, a population of tissue resident stem/progenitor cells has been recently described. Identification of methods to trigger their activity could lead to exploitation of their potential therapeutically. Here we validate the use of transgenic mice reporting cell cycle progression (FUCCI), and stemness (Lgr5-GFP), as a valuable tool to identify regulators of cell cycle re-entry of supporting cells within the auditory epithelium. The small molecule compound CHIR99021 was used to inhibit GSK3 activity. This led to a significant increase in the fraction of proliferating sphere-forming cells, labeled by the FUCCI markers and in the percentage of Lgr5-GFP + cells, as well as a selective increase in the fraction of S-G2-M cells in the Lgr5 + population. Using whole mount cultures of the organ of Corti we detected a statistically significant increment in the fraction of proliferating Sox2 supporting cells after CHIR99021 treatment, but only rarely appearance of novel MyoVIIa+/Edu + hair cells. In conclusion, these tools provide a robust mean to identify novel regulators of auditory organ regeneration and to clarify the contribution of stem cell activity.

Sound perception in mammals relies on the function of specialized mechano-sensitive hair cells located within the organ of Corti (OC). These hair cells transfer mechanical stimuli generated by the sound waves to the contacting neurons of the auditory nerve, which further relays to the auditory cortex. Loss of hair cells is a major cause of deafness worldwide. Due to the absence of an effective endogenous regenerative potential of the auditory epithelium, much effort is put into identifying strategies to preserve or to generate new hair cells^1^.

Mechano-sensory hair cells are organized in a mosaic structure with non-sensory supporting cells within the epithelium. The latter have been recently recognized as dormant stem/progenitor cells of this organ^2^,^3^,^4^,^5^,^6^. This complex tissue architecture is established during development and terminal mitoses occur as early as E12.5 in mice. By E14.5, the sensory epithelium consists of postmitotic cells^7^. Under normal physiological conditions, tissue resident stem/progenitor cells lack the capacity to re-enter cell cycle or to generate new functional hair cells. However, in specific experimental setups manipulating cell cycle inhibitors such as p27 or Rb^8^,^9^,^10^,^11^ or by altering the activity of key developmental regulators such as Notch^6^ or Wnt signaling^2^,^12^, they can be induced to proliferate and/or trans-differentiate into hair cells. Stem/ progenitor cells have been recently identified in the OC by the expression of the R-Spondin receptor Lgr5^2^,^3^,^5^,^12^,^13^. Genetic ablation of hair cells *in vivo* was shown to drive stem cell activity in the Lgr5 + cell pool, contributing to some extent to spontaneous hair cell regeneration. This occurred though, at very low levels and only in early postnatal stages^5^. Similar results were obtained after hair cell damaged with ototoxic compounds in organotypic cultures^13^.

Transgenic animal models have demonstrated in great detail how Notch and Wnt signaling control stem cell proliferation and differentiation in the OC. Translation of these findings towards therapeutic application will require identification of selective small molecule inhibitors able to induce stem cell activity by driving re-expression of positive cell cycle regulators or by triggering developmental genes.

Here, we have established and validated a platform that allows for ready detection of the rarely occurring cell cycle re-entry of otic stem/progenitor cells upon small molecule compound application. We have made use of a combination of previously described FUCCI^14^,^15^ and Lgr5-GFP reporter animals^2^,^3^,^16^ to follow the fate of otic progenitors using sphere forming assays and whole mount cultures.

The FUCCI reporter relies on the mutually exclusive expression of fluorescently tagged constructs during each cell cycle phase, and is based on the pattern of selective degradation of two proteins, Geminin and Cdt1, during G1 and S/G2/M respectively. G0/G1 cells are therefore marked by the expression of Cdt1 fused to the red fluorescent protein Kusabira Orange (Cdt1-KO2), while cells in S/G2 or early Mitosis will express Geminin, fused to the green reporter Azami Green (Gem-AG). In combination with the stem cell reporter Lgr5, the FUCCI system allows for analysis of cell cycle re-entry and progression of Lgr5 + OC supporting cells.

Our work identifies that proliferation of otic stem/progenitor cells can be triggered by a small molecule inhibitor targeting GSK3: CHIR99021. At the concentration of 10 μM, CHIR99021 was sufficient to induce the proliferation of sphere forming cells and substantially increased the percentage of Lgr5-GFP + cells. Furthermore, it specifically promoted cell cycle re-entry of Lgr5 + cells in sphere assays. Using whole organ cultures of FUCCI reporter lines we detected a significant increase in the proliferation of Sox2 + supporting cells. Finally, we have identified new but rare hair cells derived from cycling cells upon treatment with CHIR99021 in OC organotypic cultures.

This platform opens the way to screen for novel compounds which are able to trigger tissue regeneration. Translation of these findings to *in vivo* local drug delivery represents a putatively interesting therapy to counteract hearing loss.

## Results

### Cell cycle regulation of otic spheres forming cells in FUCCI transgenic reporter animals

**Figure 1:**
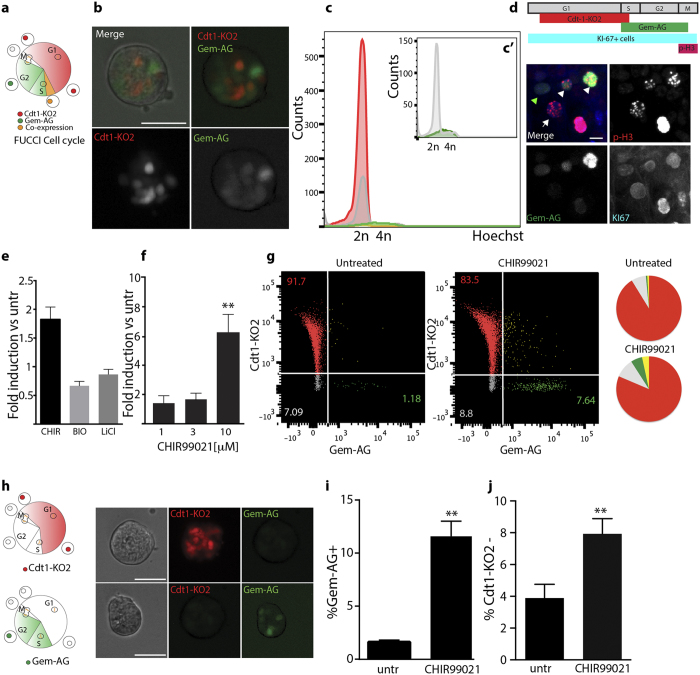
(**a**) Schematic of the FUCCI reporter. Cell cycle phases are color coded according to the fluorescent-tagged reporters. (**b**) Representative example of otic sphere from double transgenic FUCCI animals. Scale bar 50 μm. (**c**) Cell cycle profile by DNA content analysis: Hoechst staining of dissociated cells derived from 5DIV otic spheres. Red: Hoechst histogram for Cdt1-KO2+cells. Green: for Gem-AG + cells, Yellow: for Gem-AG +/Cdt1-KO2+cells, Gray: for double negative cells. c’) Re-scale Histogram from Gem-AG + cells (green) and double negative cells (Gray) as in C. (**d**) Schematic of the temporal expression of the selected markers (top) and immunostaining of cells derived from Gem-AG FUCCI animals (bottom): Gem-AG (green), Ki67 (blue) and phospho-Histone 3 (red). PH3 +/Ki67 +/Gem-AG + (early-M) cells are indicated by the white arrow heads, PH3 +/Ki67 +/Gem-AG- (late-M) cells by the white arrow. PH3-/Ki67 +/Gem-AG + (S/G2) by green arrow, Scale bar 10 μm (**e**) Fold induction of total cell number compared to control sample. Cells are treated with 10 μM CHIR99021, 0.75 μm BIO or 10 mM LiCl at day 0 and harvested at day 5 (Mean +/− SEM, Wilcoxon Paired test (ns) n = 4). (**f**) Fold induction of total cell number compared to control sample. Primary spheres were cultured for 5 days in presence of different concentrations of CHIR99021 (Mean +/− SEM **P < 0.01 Wilcoxon Paired test. (n = 3 for 1 μM, n = 3 for 3 μM and n = 9 for 10 μM and control). CHIR99021 was added at day 0 and day 3. (**g**) Flow cytometric analysis of FUCCI spheres treated with CHIR99021 for 5 days. The percentage of each population is indicated in the quadrants. (For Gem-AG + cells, the percentage reported is the sum of Gem-AG +/Cdt1-KO2- and Gem-AG +/Cdt1-KO2 + ). Pie charts show the distribution of cells in the 4 populations. Color code according to the FUCCI reporter. (**h**) Schematic of the cell cycle of Cdt1-KO2 single positive (top) and Gem-AG single positive (bottom) cells and representative example of otic spheres derived from single transgenic animals at day 5 in untreated conditions. Scale bar 50 μm. (**i**) Quantification of Gem-AG + cells (n = 4) and (**j**) Cdt1-KO2- cells (n = 11) in single transgenic lines after 5 days *in vitro* in presence/absence of 10 μM CHIR99021. (Values are Mean +/− SEM **P < 0.01 Paired t test).

In order to identify regulators of cell cycle re-entry in otic progenitor cells, we utilized a previously described reporter system: FUCCI^14^ (Fig. 1a). The organ of Corti was isolated from early postnatal (age p5) FUCCI double transgenic animals and otic spheres were generated as previously described^17^. At day 5, solid spheres^18^ were expressing both reporters, indicating proliferative activity (Fig. 1b). Flow cytometric analysis of the DNA content of day 5 otic sphere-derived cells revealed that 90% of the cells expressed the red fluorescent reporter Cdt1-KO2, indicative of G1 or G0 cells, further confirmed by Hoechst staining (Fig. 1c). Cells expressing the green fluorescent reporter Gem-AG, marking S-G2-M cells, represent 1.6 +/−0.1% of the population (Fig. 1c,c’). Cells lacking both reporters are mostly diploid (2n), (Fig. 1c’, gray histogram), suggesting that they are likely early G1 cells, which have not yet acquired red fluorescence after mitosis as previously described^15^ . A very small percentage of double negative cells with tetraploid (4n) DNA content (Fig. 1c’, gray histogram), labels late-mitotic cells. Correct expression of the reporters was further validated by immunostaining of cells for the proliferative marker Ki67, labeling all proliferative cells, independently of their cell cycle phase, and for Phospho-H3 (Ser10), marking mitotic cells (Fig. 1d). Indeed, Gem-AG + cells are a subset of the Ki67 population and include Phospho-H3 + mitotic cells (arrow heads in Fig. 1d). During transition from metaphase to anaphase, Geminin is known to be degraded^19^. Late mitotic cells therefore lack Gem-AG expression but are instead identified by positive signals for Ki67 and Phospho-H3 (arrow in Fig. 1d), as previously shown^14^,^20^.

Based on recent reports indicating that neonatal OC stem/progenitor cells are Wnt responsive, we added recombinant Wnt3a and R-Spondin 1 at different concentrations during the first 5 days of culture in non-adherent conditions. We could not detect any increase in cell number, nor in sphere forming capacity. Cells had instead increased tendency to adhere to the culture plates (Supplementary S1A and S1B). In contrast, when we tested different GSK3 inhibitors, namely CHIR99021 (10 μm), BIO (0.75 μM) and LiCl (10 mM), that through inhibition of β-Catenin degradation should result in the activation of the canonical Wnt signaling pathway, we could detect an increase in cell number (Fig. 1e). CHIR99021 appeared to be the most powerful compound to induce proliferation. Different concentrations were tested (Supplementary S1C) and 10 μM was selected for further experiments. We further optimized the culture condition and supplemented the culture at day 0 and day 3 with CHIR99021. At the concentration of 10 μM this resulted in a significant increase (6.2 +/−1.2 fold induction) in the number of cells in primary (Fig. 1f) and secondary spheres (2.54 +/−0.93 fold induction) compared to untreated samples.

Otic spheres derived from double transgenic FUCCI mice showed an increase in the proportion of S/G2/M Gem-AG positive cells after treatment with CHIR99021 and a concomitant decline of Cdt1-KO2 positive cells (Fig. 1g). This was additionally quantified also in single transgenic reporters (Fig. 1h), where the fraction of Gem-AG + cells was increased from 1.62 +/− 0.18% to 11.59 +/−1.4% after CHIR99021 treatment (Fig. 1i), while the fraction of Cdt1-KO2 negative cells increased upon treatment from 3.88 +/− 0.8% to 7.9 +/−0.9% (Fig. 1j). Inhibition of GSK3 activity using CHIR99021 has therefore a pro-proliferative effect on otic sphere forming cells.

### CHIR99021 induces cell cycle re-entry and proliferation of Lgr5-GFP + cells

**Figure 2:**
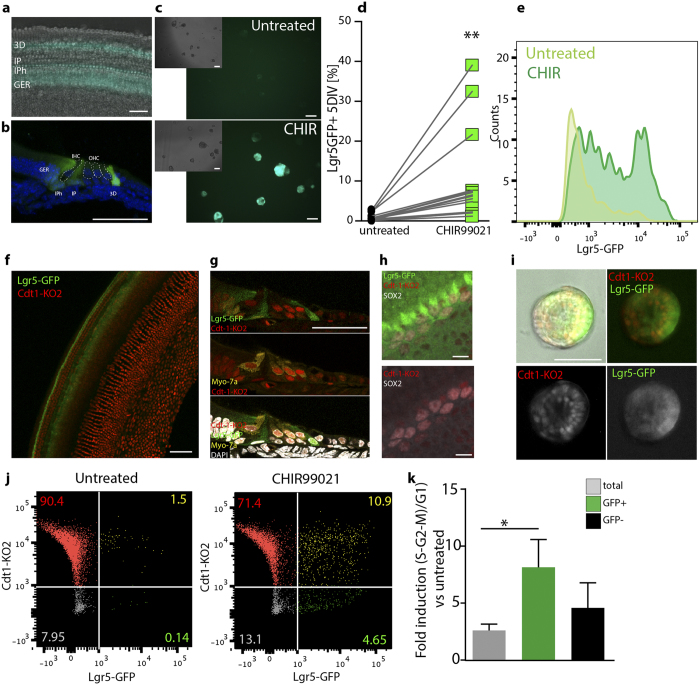
(**a**) Organ of Corti whole mount from Lgr5-GFP transgenic animals at isolation. Scale bar 50 μm (**b**) Representative image of a cryosectioned Lgr5-GFP + OC from p5 animals. Scale bar 50 μm (**c**) Otic spheres derived from Lgr5-GFP animals after 5 days in culture in presence (bottom) /absence (top) of 10 μM CHIR99021. Inserts show the bright field image. Scale bar 100 μm (**d**) Quantification of Lgr5-GFP + cells by flow cytometry from day 5 otic spheres. (**p < 0.05 Paired t test, n = 14 independent experiments) (**e**) Representative example of GFP expression analyzed by flow cytometry of otic spheres cultured for 5 days in presence/absence of 10 μM CHIR99021. (**f**) Organ of Corti at isolation from Cdt1-KO2/Lgr5-GFP animals. Scale bar 50 μm (**g**) Representative image of Cdt1-KO2/Lgr5-GFP cochlea from p5 Tg animals immunostained for MyoVIIa (yellow) and counterstained with DAPI (white). Lgr5-GFP (green), Cdt1-KO2 (red). Scale bar 50 μm. (**h**) Confocal image of Cdt1-KO2/Lgr5-GFP OC immunostained for Sox2 (white). The area selected corresponds to the 3^rd^ row of Deiters’ cells and Hensen’s cells region. Scale bar 10 μm (**i**) Otic spheres derived from double Tg animals at day 5, (untreated). Scale bar 50 μm. (**j**) Flow cytometry quantification of cell cycle profile in Cdt1-KO2/Lgr5-GFP. (**h**) Quantification of the ratio between Cdt1-KO2 negative cells and Cdt1-KO2 positive cells in the total, Lgr5-GFP + and GFP negative population. Fold induction relative to the untreated samples is shown (Mean +/− SEM * = p < 0.05 n = 5. Kruskal-Wallis test with Dunn’s correction).

Lgr5 has been recently identified as a specific marker labeling Wnt-responsive supporting cells within the OC, acting as hair cell progenitors^2^,^3^. To assess if the observed GSK3i-induced proliferation is due to stem/progenitor cell expansion, we made use of Lgr5-GFP reporter animals. At postnatal day 5 (p5), cells in the greater epithelial ridge (GER) inner pillar (IP), inner phalangeal (IPh) and the third row of Deiters’ cells (3D) express the GFP reporter (Fig. 2a,b)^2^,^3^,^21^. Otic spheres were derived from p5 OC and expanded for 5 days *in vitro* in presence or absence of CHIR99021 (Fig. 2c). Treatment with 10 μM CHIR99021 at day 0 and day 3 resulted in a robust increase in the fraction of GFP positive spheres (Fig. 2c). This was further quantified by flow cytometry (Fig. 2d and Supplementary S2 C), where GFP positive cells were analyzed after primary sphere dissociation. While at isolation GFP + cells represented 6.9 ± 1.8% of the culture (Supplementary S2 C), in agreement with previously published data^2^,^3^, this proportion dramatically decreased in culture. At day 5, only 0.9% +/−0.2 of cells expressed detectable amounts of GFP. In contrast, in presence of CHIR99021, the percentage of GFP positive cells was significantly increased, reaching a mean value of 10.8% +/− 3.1 (range from 2.2% to 39.1% over 14 different experiments as shown in Fig. 2d), leading to an overall fold increase in the number of GFP + cells of 14.14 +/− 2.1 compared to untreated cells. Concomitantly, we detected a significant increase in sphere size (Supplementary S2 D) and increased mean GFP signal intensity (Fig. 2e). Treated cells had 2.8 +/−0.7 fold higher intensity, assessed by flow cytometry. This is in agreement with the fact that Lgr5 itself is a target of canonical Wnt signaling. Additionally, Sox2 was found to be upregulated in CHIR99021 treated spheres (Supplementary S2E,F).

In contrast, we could not detect any increase in the percentage of GFP + cells after treatment with recombinant Wnt3a and R-Spondin1 at all concentration tested (Supplementary S2A,B,C).

In order to label the cell cycle of Lgr5 + cells with the FUCCI system, we crossed Cdt1-KO2 reporter mice with Lgr5-GFP transgenic animals (Fig. 2f). We analyzed the sensory epithelium of double transgenic Cdt1-KO2/ Lgr5-GFP mice by immunostainings for Sox2 and MyoVIIa, either in whole mount preparations or in cryosections. Both, hair cells as well as supporting cells (Lgr5 + or Sox2 + ) expressed the G1/G0 reporter Cdt1-KO2, in agreement with their quiescent state (Fig. 2f–h; see also Supplementary S3 B and C). We then performed flow cytometry analysis directly after dissection and single cell trituration of the tissue. Lgr5-GFP +/Cdt1-KO2 + double positive cells represent 5.53% +/−0.4 of the cell population, in agreement with the percentages obtained from single Lgr5-GFP animals. Cells were then cultured as otic spheres for 5 days (Fig. 2i) and the percentage of Lgr5-GFP positive cells, negative for Cdt1-KO2 expression was used as a readout for cell cycle re-entry, more specifically for cells in early G1 or S-G2-M phase. We quantified the relative percentages of cells in different cell cycle phases by flow cytometry upon treatment with CHIR99021 (Fig. 2j,k). In the total population, CHIR99021 induced a 2.6-fold increase in the ratio of (Cdt1-KO2-)/ Cdt1-KO2+cells compared to untreated cells. In contrast, the greatest induction (8.1 folds +/− 2.4) was observed for the Lgr5-GFP + population where 20.54% +/−4.4 of the GFP + cells were found in cycle (Cdt1-KO2 negative) after CHIR99021 treatment. These results overall support the idea that the GSK3 inhibition leads to expansion of the Lgr5-GFP cell pool and cell cycle progression. However, its effect is not limited to these cells.

### CHIR99021 treatment does not affect the differentiation efficiency towards hair cells

**Figure 3:**
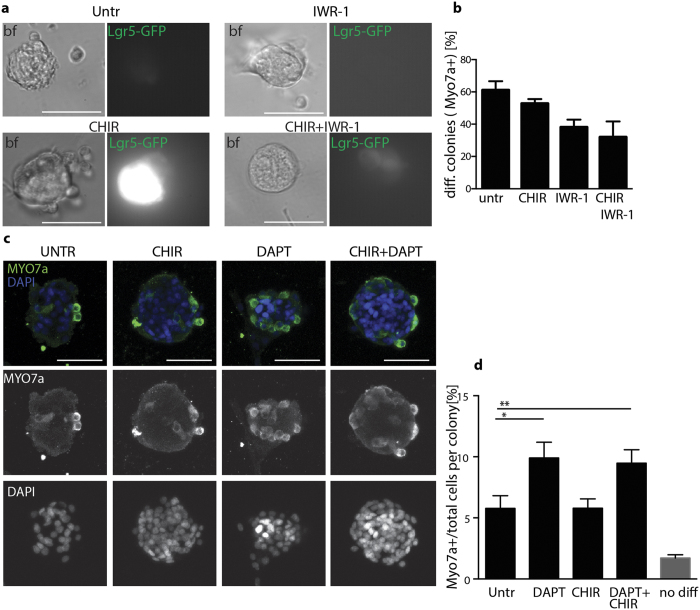
Representative images of OC spheres at day 5 *in vitro* cultured in presence of CHIR99021 (10 μM), IWR-1 (2.5 μM) or the combination of the two. Brightfield (left) and GFP (right) channels are shown. Scale bar 50 μm. (**b**) Quantification of the fraction of colonies containing MyoVIIa + cells over the total colonies counted (n = 20 from 2 independent experiments) after 15 days of differentiation. CHIR99021 and IWR-1 are added exclusively during the first 5 days in sphere culture. (**c**) Representative examples of spheres after differentiation immunostained for MyoVIIa (green) and DAPI (blue). Cells are treated with/without CHIR99021 in sphere cultures and further plated for differentiation with/without DAPT (1 μM) for additional 15 days. Maximum intensity Z projections of confocal stacks are shown as merged and single channels. Scale bar 50 μm. (**d**) Quantification of the percentage of MyoVIIa positive cells per colony. Cells are treated with/without CHIR99021 in sphere culture and further plated for differentiation with/without DAPT (1 μM) for additional 15 days. Mean +/− SEM *p < 0.05 (Kruskal-Wallis test with Dunn’s correction). 70–80 spheres are counted per condition and derived from 7 independent experiments. Untreated cells (primary spheres) at day 5 *in vitro* are plated in parallel and after 1 day fixed for immunostaining. MyoVIIa + cells are counted prior to differentiation to estimate carried over hair cells from the isolation (gray bar). All samples are statistically significantly different from negative control. (Kruskal-Wallis test with Dunn’s correction).

Previous studies have shown that excessive Wnt signaling activation would impair differentiation capacity of Lgr5/Sox2 precursor cells^12^. Treatment with small molecule compounds offers the advantage of transiently activating the pathways at specific time points. We therefore set out to assess the differentiation capacity of spheres where Wnt signaling had been chemically modified. To this end, primary spheres cultured for 5DIV in presence of either CHIR99021 and/or the Wnt antagonist IWR-1, were seeded in growth factor depleted medium for additional 15 days to induce differentiation. Treatment with IWR-1, known to induce accumulation of Axin2 and β-catenin phosphorylation^22^, resulted in a strong decrease in the expression of GFP from Lgr5-GFP cells and abolished the CHIR99021-mediated induction of GFP expression (Fig. 3a). Moreover, this resulted in a reduction in the number of MyoVIIa + colonies (Fig. 3b), suggesting that IWR-1 treatment has affected either the number or the capacity of cells initially expressing Lgr5 to differentiate to hair cells. CHIR99021 in contrast, did not affect the fraction of differentiated spheres.

Inhibition of Notch signaling using γ secretase inhibitors, has been previously shown to induce hair cell differentiation^23^,^6^. We therefore examined the percentage of MyoVIIa + cells per colony obtained after plating primary spheres in presence of the γ secretase inhibitor DAPT. DAPT was added on day 6, after spheres had adhered, to induce differentiation (Fig. 3c). Quantification of the number of hair cells per sphere at day 15 shows that CHIR99021 treatment is not affecting both the basal as well as the DAPT induced differentiation (Fig. 3c,d).

### CHIR99021 induces cell cycle re-entry of supporting cells in organotypic cultures

In order to assess if cell cycle re-entry could be also detected in whole mount organ cultures, where cell-cell contacts and lateral inhibitions are still preserved, we established explant cultures from FUCCI transgenic animals, specifically from Gem-AG single transgenics. Here, Induction of proliferation can be easily assessed by the appearance of the S-G2-M reporter. At isolation the entire OC lacks in fact green fluorescence, in agreement with its quiescent and postmitotic state (Supplementary S3A).

**Figure 4:**
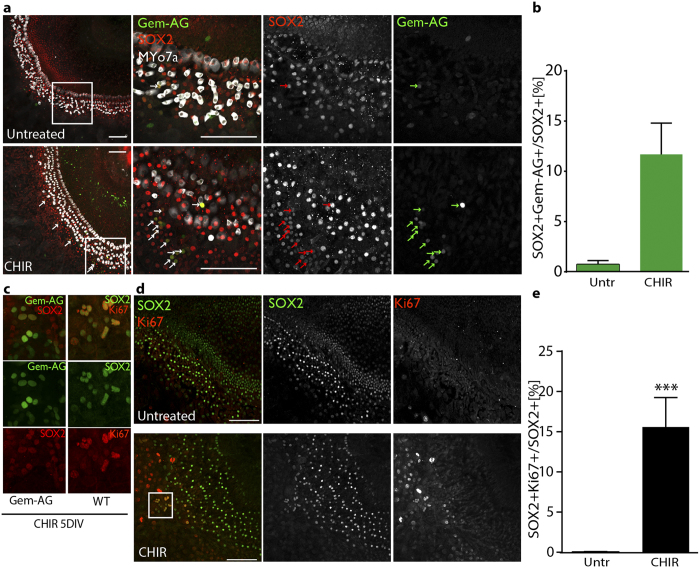
(**a**) Confocal imaging (maximum Z stack projections) of organotypic cultures of FUCCI Gem-AG organ of Corti (5 days *in vitro*) from p5 animals, (Middle turn regions are shown) untreated or CHIR99021 treated. Merge and single channels are displayed: Gem-AG (green), MyoVIIa (white), Sox2 (red). Arrows indicated double positive cells. Scale bar 100 μm (**b**) Quantification of double positive (Gem-AG +/Sox2 + relative to the total Sox2+cells counted). OC from 3 animals were imaged by confocal microscopy. 10 different fields were imaged from basal to apex per OC and a total number of ca. 2000 Sox2+cells was counted. Values are Mean +/− SEM (**c**) Higher magnification of double positive cells Gem-AG +/Sox2 + (left) or Ki67/Sox2 + (right). (**d**) Confocal imaging (maximum Z stack projections) of organotypic cultures of WT organ of Corti (5 days *in vitro*), from p5 animals (Middle turn regions are shown) untreated or CHIR99021 treated. Merge and single channels are displayed: Ki67 (red), Sox2 (green). Scale bar 100 μm. White boxed area is enlarged in 4c (**e**) Quantification of double positive cells (Ki67 +/Sox2 + relative to the total Sox2+cells counted). OC from 5 animals (age p5) were imaged by confocal microscopy. 10 different fields were imaged from basal to apex for each OC and a total number of ca. 6000 Sox2 cells was counted per condition. Values are Mean +/− SEM ***p < 0.001 Wilcoxon paired test.

Within the organ of Corti, visualized by staining the cultures at day 5 for Myo-VIIa and Sox2, we identified Gem-AG + cells almost exclusively in the CHIR99021-treated samples (Fig. 4a,c). The double positive Sox2 + and Gem-AG + cells were quantified relative to the total number of Sox2+cells counted. In presence of CHIR99021, this reached 11.7 +/−3.12% (Fig. 4b). Similar results were obtained using Cdt1-KO2 mice, where we quantified the number of Sox2+cells that lacked the expression of the G0/G1 reporter (Supplementary S3 B,C). Finally, we could confirm this by immunostaining of OC cultures from wildtype animals for the proliferative marker Ki67 (Fig. 4c–e). Also in this case, only in the CHIR99021 treated samples, proliferation was observed within the OC. Here, the percentage of Ki67 +/Sox2+cells increased to 15.6% +/−3.6 (Fig. 4e). In all cases, proliferative Sox2+ cells were found to be mostly located in the area of Hensens’ and 3^rd^ row of Deiters’ cells, however due to the substantial outgrowth of the tissue upon CHIR99021 treatment, it is difficult to attribute cell identity with accuracy. Proliferation was furthermore observed at all cochlear regions (Supplementary Figure S4).

Overall, the CHIR99021 treatment induces a significant increase of Sox2 proliferative cells also in cultured OC whole mounts.

### New MyoVIIa + hair cells are formed upon treatment with CHIR99021 in organotypic cultures

To assess if the induction of proliferation in the OC would further lead to generation of new hair cells, we incubated organotypic cultures with the thymidine analog Edu and analyzed the cultures at day 5 for the presence of immunoreactive MyoVIIa + cells that had incorporated Edu, and therefore undergone DNA synthesis.

**Figure 5:**
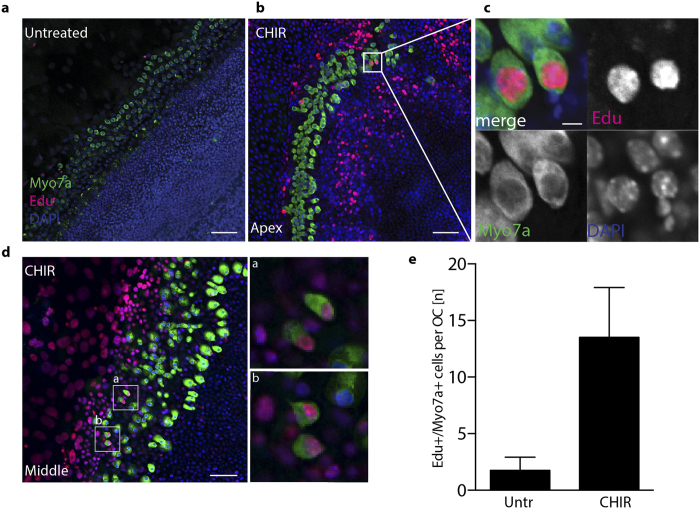
(**a**) Confocal imaging (maximum Z stack projections) of organotypic cultures from Wt (p3) animals incubated 5 days *in vitro* with 5 μM Edu (Apical turn). Samples are stained at day 5 for MyoVIIa (green) and Edu (red). Nuclei are visualized by DAPI staining (blue) Scale bar 50 μm. (**b**) Organotypic culture from Wt (p3) animals incubated for 5 days *in vitro* in presence of 10 μM CHIR99021 and 5 μM Edu. (Apical turn). (**c**) Higher magnification of the white-boxed area in b. Single channels are shown for double positive hair cells. Scale bar 10 μm. (**d**) Representative image of organotypic OC cultures from wt animals (p3), (Middle turn). Scale bar 50 μm. Selected areas (white boxes, are enlarged in a and b). (**e**) Quantification of the average number of MyoVIIa+/Edu + cells per OC after CHIR99021 treatment in p3 animals (n = 4).

We could indeed detect double positive cells in samples treated with CHIR99021. The number of MyoVIIa+/Edu+cells was however limited: 13.5 +/−4.4 new hair cells per OC (Fig. 5). Double positive cells were mostly restricted to the apical (40%) and middle turn (60%) (Supplementary S4A and S4B). No double positive cells have been observed in the basal turn, despite the proliferation of Sox2 cells along the entire OC (Supplementary Figure s4c). Similar experiments were performed in Cdt1-KO2 animals (Supplementary Figure S5). Here, MyoVIIa + cells lacking Cdt1-KO2 expression could be identified upon CHIR99021 treatment. However, only very few cells per OC have been detected (2.25 +/−1.43), suggesting that direct proliferation of hair cells account for a small part to the newly formed Edu +/MyoVIIa + cells in the CHIR99021 treated samples.

In conclusion, while GSK inhibition leads to a robust expansion of the stem/progenitor cell pool, differentiation and maturation of the newly formed cells may not be fully supported in *ex-vivo* organ culture and may require prolonged culture timing or alternative cues.

## Discussion

The aim of this study was to establish and validate a platform to easily and reliably detect cell cycle re-entry of dormant/quiescent cells within the OC. To this end, we have been making use of a combination of cell cycle reporters (Gem-AG and Cdt1-KO2) and Lgr5-GFP reporter mice to assess the proliferative capacity of Lgr5+ and Sox2+cells from the OC.

The role of Wnt signaling in the expansion of the Lgr5 + progenitor pool has been recently described^2^,^3^,^12^. Agonists of the canonical Wnt signaling pathway were therefore selected in order to validate our system. Recombinant Wnt3a, alone or in combination with the Lgr5 ligand R-Spondin as well as different GSK3 inhibitors, were initially tested for their efficacy to induce proliferation in sphere assays in Wt and FUCCI transgenic animals.

Using this approach we have successfully identified CHIR99021 as the strongest small molecule inhibitor, significantly increasing proliferation in the organ of Corti both in primary and secondary sphere assays. Interestingly, CHIR99021 also led to a significant increase in the fraction of Lgr5-GFP positive cells. While the Lgr5 expressing cells seem to be the most affected by the treatment, all cells in the sphere culture are stimulated to proliferate (as shown in Fig. 2k).

Treatment with CHIR99021 resulted in a more robust induction of proliferation and Lgr5-GFP expression compared to Wnt3a and R-Spondin supplemented as recombinant proteins as previously reported^2^,^3^ (Supplementary S1 and S2). Primary versus second-generation spheres^3^ and prospectively sorted Lgr5 cells as starting point for these assays^2^ may explain the small discrepancies observed between these studies and our.

The effect of GSK3 inhibition on proliferation of the cochlear epithelium was demonstrated using embryonic explant cultures and treatment with the small metal cation LiCl^24^. Here, we demonstrate that this occurs also in early postnatal stages using instead an ATP-competitive GSK3 inhibitor. CHIR99021 was previously shown to be required for mESC self- renewal as well as to enhance reprogramming efficiency^25^,^26^,^27^. The mechanisms by which GSK inhibition helps to maintain self-renewal has been attributed to both the positive role exerted on metabolic activity, biosynthetic capacity and overall cell viability, as well as to it’s effect on TCF3 repression^26^. Therefore, the effects observed in our culture on expansion of the stem/progenitor cell pool may not solely be ascribed to the effect of Wnt signaling but also to a synergy between the metabolic action of GSK3 and it’s role in controlling the pluripotency gene network. Further experiments, looking at global gene expression changes in the Lgr5 population may help to dissect further the relative contribution of each of the pathway/processes affected through GSK3 inhibition.

The use of small molecule inhibitors offers the possibility of only transiently activating/inhibiting a certain pathway. For example, genetic deletion of exon 3 of the β-catenin gene, causing constitutive activation of canonical Wnt signaling, resulted in decreased capacity of Lgr5 cells to differentiate to MyoVIIa + cells in a dose dependent manner^12^. In our hand, GSK3 inhibition was limited to the expansion phase during sphere cultivation. Upon removal of the compound, we did not observe any significant impairment in the capacity of the Lgr5 + cells to differentiate to hair cells neither in basal, nor DAPT-induced conditions. Beside Lgr5 itself (as shown in Fig. 2e) also Sox2 was found to be strongly up-regulated upon CHIR99021 treatment (Supplementary S2). It is therefore possible that during the differentiation phase, supporting cell identity also gets specified, at the expenses of hair cell fate. This may explain why despite the increase in the progenitor pool, the percentage of hair cell like cells per colony remains the same.

Elegant studies using Lgr5-GFP sorting strategies have previously shown that these cells are capable to differentiate into hair cells *in vitro*^2^,^3^. Here we assessed differentiation efficiency in unsorted cell populations using primary spheres. This was done in view of establishing a compound screening method, based on the FUCCI and Lgr5 reporters, suitable for up-scaling to medium/high throughput, therefore capable to pick up differences in proliferative and differentiation capacity over background. This was in fact the case for the strong pro-proliferative effect exerted by CHIR99021 as well as for the Notch-inhibitor induced differentiation as previously reported^23^.

Using organotypic cultures we have been able to show that treatment with CHIR99021 induces proliferation in intact organs. We detected a statistically significant increase in the number of Sox2 + supporting cells co-labelled with the proliferative marker Ki67 or Gem-AG, or negative for Cdt1-KO2, overall going from 0.6% +/−0.4 in untreated condition to 14.6% +/−2.9 after CHIR99021 treatment.

Lgr5+ cells represent a subpopulation of the Sox2 expressing supporting cells. We attempted to monitor cell cycle re-entry of Lgr5 cells using Lgr5-GFP/Cdt1-KO2 reporters, however, we were unable to rely on endogenous GFP expression for quantification. This was due to low levels of GFP expression after 5 days *in vitro*, together with the change in morphology of supporting cells, making impossible to unambiguously attribute cell identity. We therefore relied on the more robust identification of Sox2 positive nuclei for this set of experiments. Due to the substantial outgrowth of the tissue upon CHIR99021 treatment, and consequent tissue disorganization, we have not quantified proliferation in different subpopulations of supporting cells. Proliferative Sox2+ cells were however found in most cases located in the area where Hensens’ cells and 3^rd^ row of Deiters’ cells reside. To be noted that only the latter expresses Lgr5. Therefore, in organ culture the proliferative response may not be predominant in the Lgr5 subpopulation as seen in sphere cultures.

Despite the fact that proliferation was observed at all cochlear regions (Supplementary S4), the newly generated Myo7a+/Edu + cells were identified exclusively in apical/middle turn regions (Supplementary S4 and Fig. 5) as recently reported by others using Notch inhibitors^6^. New MyoVIIa positive cells arose exclusively in samples treated with CHIR99021. However, the number of these cells was rather low, suggesting that different cues must be required in order to induce cell differentiation and maturation at least in this specific experimental set up *in vitro*.

Our study made use of early postnatal mice ranging between 3 and 5 days after birth. Recent reports have demonstrated that in earlier time points this effect may be more pronounced^5^. More studies will be required in order to dissect the time dependency of these events. However, in view of exploiting these findings as possible hearing loss therapy, we believe it would be of higher relevance to assess the efficacy of these compounds in adult animals.

While genetic tools provide the possibility to identify novel regulators of stem cell activity, translation towards clinical application will rely on the identification of very selective small molecule compounds able to trigger the activation/inhibition of these pathways. This approach was recently successfully pioneered using γ-secretase inhibitors, targeting the Notch signaling pathway^6^,^23^. Combination of Wnt signaling activation and Notch signaling inhibition may therefore provide a more robust induction of tissue regeneration *in vivo*.

In conclusion, the combination of FUCCI and stem cell reporters provides a rapid and reliable way to identify activators of cell cycle re-entry and screen for small molecule compounds exerting this function on the tissue resident stem cell pool.

## Material and Methods

### Animals

Mice containing an EGFP-IRES-CreERT2 “knock-in” allele at the *Lgr5* locus^16^, referred to as Lgr5-GFP were obtained from Jackson Labs (Stock 008875). FUCCI animals: B6.Cg-Tg (Fucci) 504Bsi (RBRC02706) [Fucci-S/G2/M-Green: mAG-hGeminin (1/110)] and B6.Cg-Tg (Fucci) 596Bsi (RBRC02707) [Fucci-G1-Red: mKO2-hCdt1 (30/120)] were obtained from the Riken institute, Japan. FUCCI line were kept as single transgenic and as heterozygotes and crossed to obtain double transgenic for experimental purposes. All mouse experiments were approved by the Animal Research Ethics Committee of the Canton Berne, Switzerland, (permission number BE117/12 and BE119/12), and were carried out in accordance with the approved guidelines.

### Dissection

Early postnatal animals, (age p3–p5) C57BL/6N background were decapitated and temporal bones were dissected out and placed in ice cold HBSS (Life Technologies) for further microdissection under a stereomicroscope. The inner ear was isolated and the cochlea opened using fine tweezers (Dumont n5, WPI). The organ of Corti (OC) and Stria Vascularis (SV) were first dissociated from the Spiral Ganglion (SG), followed by the removal of the SV.

### Sphere formation and Flow cytometry analysis

For cell isolation/sphere culture previously published methods^17^,^28^ were followed with slight modifications. Specifically, for each animal, 2 OC were placed in a 50 μl drop of trypsin/EDTA 0.025%, (Life Technologies) at room temperature (RT) for a couple of minutes and cut in smaller pieces using forceps under a stereomicroscope. 50 μl of 0.25% Trypsin/EDTA was then added to each drop and incubated at 37 °C for 10 minutes in a cell culture incubator. Immediately after, 50 μl of Trypsin inhibitor/DNAseI cocktail (both at 1 mg/ml, Sigma) and 50 μl of culture medium were added to each OC at RT. The tissue was further triturated by gentle pipetting with a 200 μl pipet tip, till obtaining a dissociated culture. Cells were resuspended in culture medium and passed through a 40 μm cell strainer (Falcon) to obtain single cell suspension. Culture medium for sphere expansion included DMEM-F12 supplemented with B27, N2 (all from Life Technologies), 20 ng/ml EGF, 10 ng/ml bFGF, 50 ng/ml IGF and 50 ng/ml Heparan Sulfate (Sigma Aldrich). For all sphere-formation assays animals were used at postnatal day 5 (p5).

For initial screening with GSKi inhibitors, OCs from different animals were pooled after trituration, passed through a 40 μm cell strainer and re-distributed in different wells so that the total cells isolated from one OC were plated in one well of a 24 well plate in 1 ml of medium. Compounds were added at day 0 and the medium was not changed for the first 5 days of culture. The following concentrations were used: CHIR99021 (Merck/Millipore) 1–10 μm, BIO (Sigma), 0.75 μM, LiCl (Calbiochem), 10 mM, IWR-1 (Merk/Millipore) 2.5 μM. 0.1% DMSO was used as control. Wnt3a (20 ng/ml, 40 ng/ml and 80 ng/ml) and R-Spondin 1 (250 ng/ml or 500 ng/ml), (both from R&D), were added to the medium at day 0. Untreated cells served as controls. The selected concentrations were based on previous reports^3^,^24^.

Alternatively, the cells from 2 OC were plated in one well of a 6 well plate, in 2 ml of medium, cultured for 2 days and re-supplemented at day 3 with additional 2 ml of medium.

At day 5 (5DIV) cells were harvested, trypsinized, counted with a hemocytometer and further passaged.

For flow cytometry analysis of GFP expression, spheres were triturated as above, passed through a 40 μm cell strainer and analyzed using a LSRII-SORP cytometer (BD). Doublets were excluded from the analysis. For flow cytometry analysis of cell cycle phases, primary spheres (5DIV) were incubated with Hoechst (Invitrogen) diluted 1:2000 in culture medium for 30’ prior to dissociation. Further processed as above. Analysis was performed offline using FlowJO (Tree star inc.).

### Differentiation

5DIV otic spheres were plated on growth factors depleted Matrigel^TM^ (Corning) coated 96 well plates. Matrigel^TM^ was diluted 1:10 in culture medium, dispensed into the bottom of the wells and incubated for 30 min at 37 °C prior to cell seeding. Cells were plated in conditioned medium (i.e. culture medium in which they were growing). All spheres derived from one OC were divided over 2 wells. Spheres were left to adhere for 2 days, after which the medium was diluted 1:2 with basic medium (DMEM-F12 + B27 + N2). 2 days later, the medium was entirely changed to basic medium. Cells were kept to differentiate for 15 days. Medium was changed every 3 days. The γ secretase inhibitor DAPT (Sigma) was added at day one after plating spheres for differentiation at the concentration of 1 μM.

At day 15, cells were fixed in 4%PFA and immunostained for Myo7a + . Spheres were randomly selected based on DAPI channel and imaged by confocal microscopy. Presence-absence of Myo7a immunoreactivity was assessed as well as the percentage of Myo7a + cells per each sphere. As control for the differentiation protocol, cells were plated in the same conditions and left to adhere for 1 day. Subsequently, cells were fixed in 4%PFA and the frequency of Myo7a + cells in primary spheres, prior to differentiation was assessed.

### Organotypic culture

p3 and p5 animals were used for organotypic cultures (see figure legends for specific age). Protocols were adapted from^13^ and^29^, with the following modification: Glass coverslips were coated with a 20 μl drop of CellTak (Corning) diluted 1:6 in DMEM-F12. OC and SV were dissected as above in HBSS. The organ of Corti was then plated on the coated coverglasses and cultured in 1 ml of full culture medium, supplemented with 10% FBS, for 5 days in 24 well plates. At day 3, 1 ml of fresh medium was added to the culture. At day 5, cultures were rinsed once in PBS prior to fixation.

CHIR99021 was added at day 0 at the final concentration of 10 μM. In experiments where 5-ethynyl-2´-deoxyuridine (Edu) (Life Technologies) was used, 5 μM Edu was added at day 0, and left during the entire culture time.

### Immunofluorescence

Samples were fixed with 4% paraformaldehyde for 10 min at RT and rinsed with PBS, permeabilized with 0.1% Triton-X100 for 5 minutes and then blocked with blocking solution (4% BSA + 0.01% Triton-X100 in PBS) for 2 h. The primary antibodies were diluted 1:100 in blocking solution (rabbit polyclonal anti MyoVIIa (Proteus); mouse monoclonal anti Sox2 (Millipore); rabbit polyclonal anti Ki67 (Novocastra), rabbit polyclonal anti Sox2 (Invitrogen) and left over night at 4 °C. The samples were then rinsed in PBS and the secondary antibody, (AlexaFluor conjugated, Invitrogen) was incubated for 2 h 1:500 diluted, in blocking solution at RT. Edu detection was performed by Click-iT (Life Technologies) detection according to manufacturer’s instructions. Samples were mounted using Fuoroshield with DAPI (Sigma). The images were acquired with a confocal microscope (Zeiss LSM 700) using a 20X air objective or 63X oil objective. Alternatively using a fluorescence microscope (Leica DMI4000) equipped with a CCD camera (Leica Microsystems).

### Image Analysis

The open source image processing software FIJI (ImageJ vesion 2.0) was used for assessment of sphere size, and signal intensity. For signal intensity quantification, 8bit images were thresholded to identify pixel + area (Sox2 specific signal) and mean gray intensity, from which integrated density was calculated. Values were further corrected for colony size, manually selected on the DAPI channel.

### Statistical analysis

#### Statistical analyses were performed using Graph Pad Prism software (version 6.0.)

To compare data between two groups, the paired *t* test was used for parametric data; otherwise, the Wilcoxon matched-pairs signed-rank test was used. For multiple comparisons, one-way analysis of variance (ANOVA) with Bonferroni correction was used for parametric data and Kruskal-Wallis test with Dunn’s correction for non-parametric data.

## Additional Information

**How to cite this article**: Roccio, M. *et al.* Cell cycle reactivation of cochlear progenitor cells in neonatal FUCCI mice by a GSK3 small molecule inhibitor. *Sci. Rep.* **5**, 17886; doi: 10.1038/srep17886 (2015).

## Supplementary Material

Supplementary Information
