# Supplementary material for: Cell cycle reactivation of cochlear progenitor cells in neonatal FUCCI mice by a GSK3 small molecule inhibitor
Source: Sci Rep. 2015 Dec 8;5:17886. doi: 10.1038/srep17886 (PMC4672274; doi:10.1038/srep17886)
Supplement: Supplementary Information [file srep17886-s1.pdf]

SUPPLEMENTARY MATERIAL

Cell cycle reactivation of cochlear progenitor cells in neonatal FUCCI mice by a GSK3 small molecule inhibitor.

Roccio M.[1*], Hahnewald S.[1], Perny M.[1,3], Senn P.[1,2]

1. Laboratory of Inner Ear Research, Department of Clinical Research, University of Bern and University Department of Otorhinolaryngology, Head & Neck Surgery, Inselspital, Bern, Switzerland.

2. Department of Otorhinolaryngology, Head & Neck Surgery University Hospital Geneva (HUG), Switzerland.

3. Laboratory of Neuroinfectiology, Institute of Infectious Diseases (IFIK), University of Bern.

Corresponding author: Marta Roccio PhD

Inner Ear Research Laboratory

Department of Clinical Research

Murtestrasse 50

3008CH Bern

Switzerland

Tel: +41 31 6327619

Email: marta.roccio@dkf.unibe.ch

Supplementary Figures Roccio et at. “Cell cycle reactivation of cochlear progenitor cells in neonatal FUCCI mice by a GSK3 small molecule inhibitor”

Supplementary Figure S1

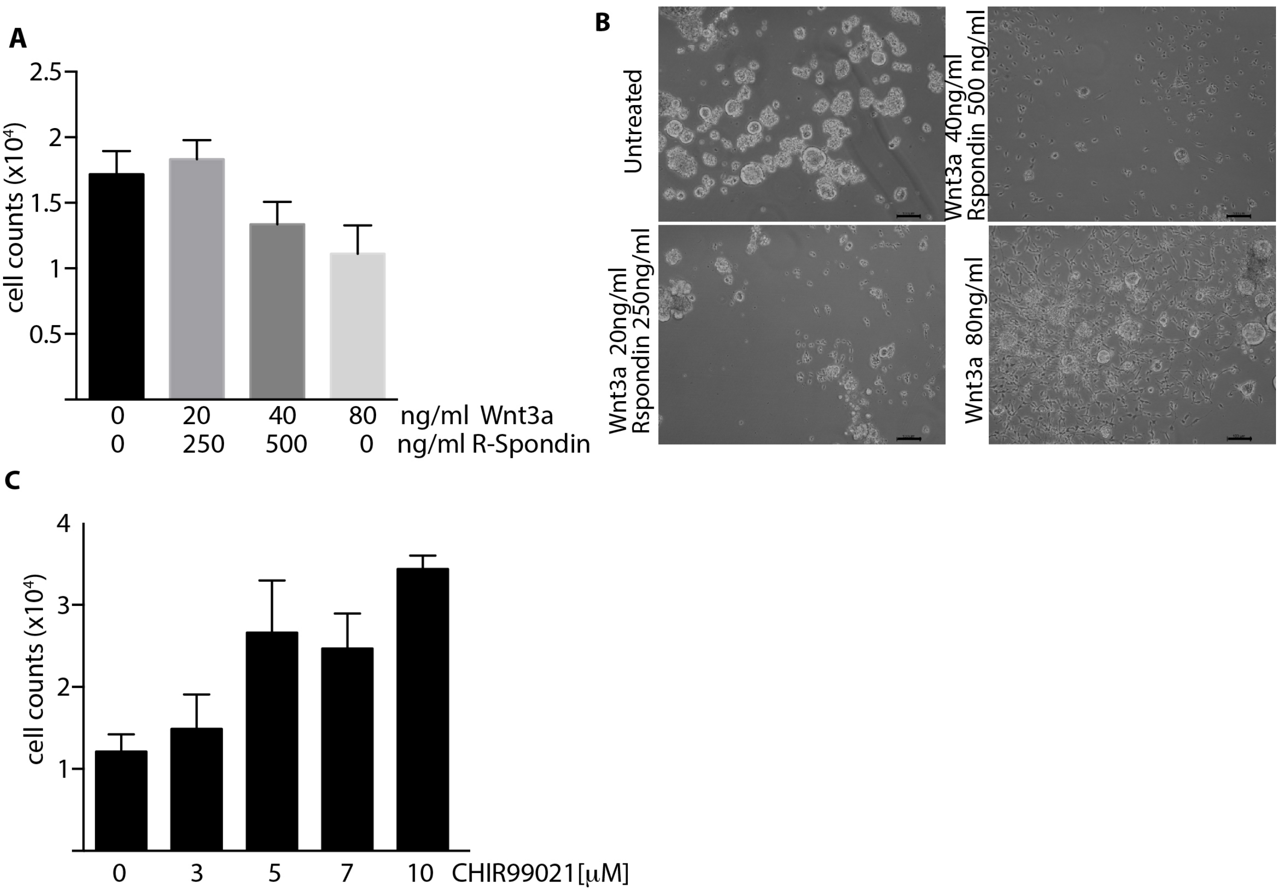

Supplementary S1 Legend

a) Quantification of total cell number after primary sphere trituration at day 5 *in vitro*. OC derived sphere forming cells were treated with different concentrations/combinations of Wnt3a and R-Spondin1 as indicated. OC from n=12 (age p5) animals were pooled after initial trituration and re-distributed over 12 wells of a 24 well plate. Recombinant proteins were added at day 0. Triplicate samples were analyzed per conditions. b) Representative brightfield images of the spheres at day 5 treated with different concentrations/combinations of Wnt3a and R-Spondin1 as indicated. Scale bar 100μm

c) Quantification of total cell number after primary sphere trituration at day 5 *in vitro*. OC derived sphere forming cells were treated with different concentrations of CHIR99021 as indicated.

OC from n=7 (age p5) animals were pooled after initial trituration and re-distributed over 15 wells of a 24 well plate. CHIR99021 was added at day 0. Triplicate samples were analyzed per conditions. 0.1% DMSO was used as control.

Supplementary Figure 2

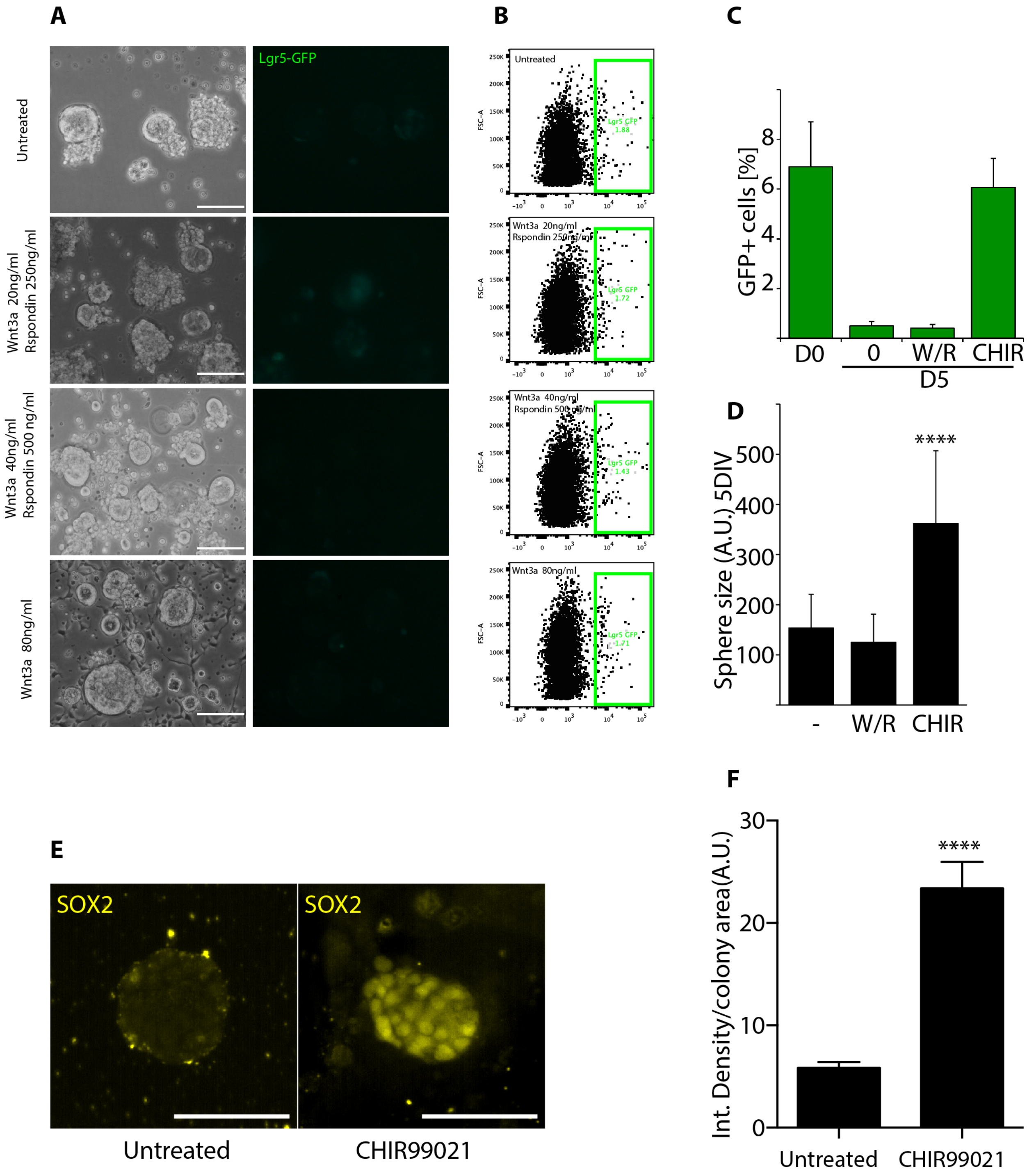

Supplementary S2 Legend

Representative images of 5DIV spheres isolated from Lgr5 GFP animals (age p5), treated with different concentrations/combinations of Wnt3a and R-Spondin1 as indicated. Brightfield and GFP channels are shown. Scale bar 100μm. B) Representative example of GFP+ cells quantification by flow cytometry for the indicated treatment at 5DIV c) Quantification of GFP+ cells by flow cytometry after 5DIV in untreated samples, Wnt3a (20ng/ml)/R-Spondin1(250ng/ml) (W/R) treated or CHIR99021 treated (10μM) cells. Comparison with GFP+ percentage at day 0 is displayed. (n=3 independent experiment). d) Quantification of sphere size after W/R (Wnt3a (20ng/ml) /R-Spondin1(250ng/ml)) or CHIR99021(10μM) treatment at 5DIV.**** $p<0.001$ ANOVA multiple comparison. e) Representative example of untreated or CHIR99021 treated spheres plated on Matrigel^{TM} coated plates after 5DIV and immunostained for SOX2. Scale bar 50μm. f) Quantification of the integrated density (pixel positive area x mean intensity), corrected for sphere area is plotted. 50 spheres from 2 independent experiments were analyzed for CHIR treatment; 35 spheres from 2 independent experiments for neg control. (*Unpaired t test)

Supplementary Figure 3

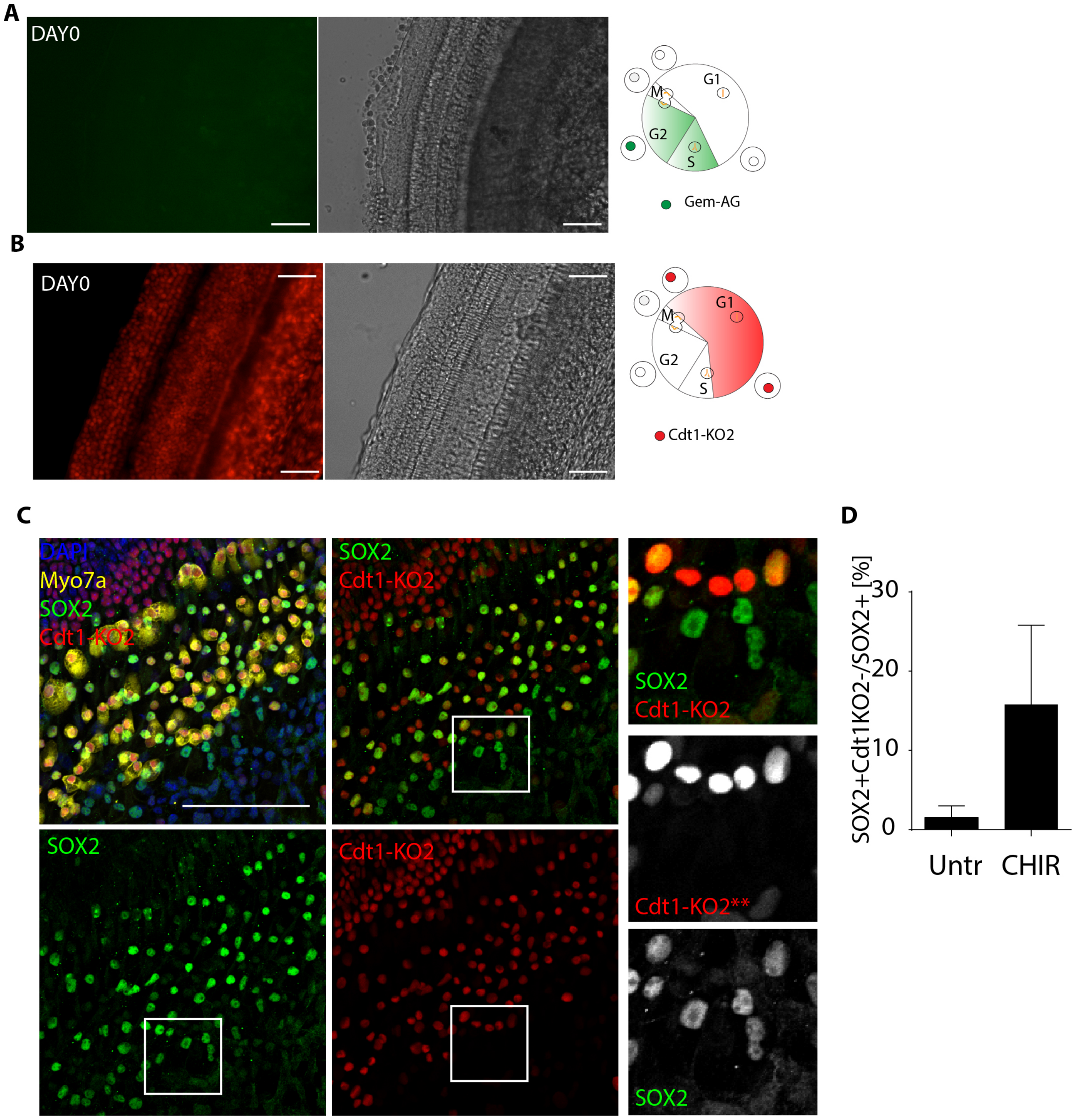

Supplementary S3 Legend

a) Organotypic culture from Gem-AG FUCCI OC at day 0 and schematic of the reporter. Fluorescence microscopy shows lack of green fluorescence at the moment of isolation. Scale bar 50μm. b) Organotypic culture from Cdt1-KO2 FUCCI OC at day 0 and schematic of the reporter. Scale bar 50μm. c) Representative example of organ cultures, 5DIV, immunostained for Myo7a (yellow), Sox2 (green). Cdt1-KO2 (red) and DAPI (blu). Merged and single images are shown (middle cochlear turn). White boxed area is shown in right panels. Cdt1-Ko2**: oversaturated image to illustrate lack of nuclear fluorescence. Scale bar 100μm. d) Quantification of Cdt1-KO2-/Sox2+ cells in Organotypic culture. OC from 3 animals (age p5) were imaged by confocal microscopy. 10 different fields were imaged from basal to apex per OC and a total number of ca. 2000 Sox 2 cells was counted. Average +/- SEM is shown in the bar graph.

Supplementary Figure 4

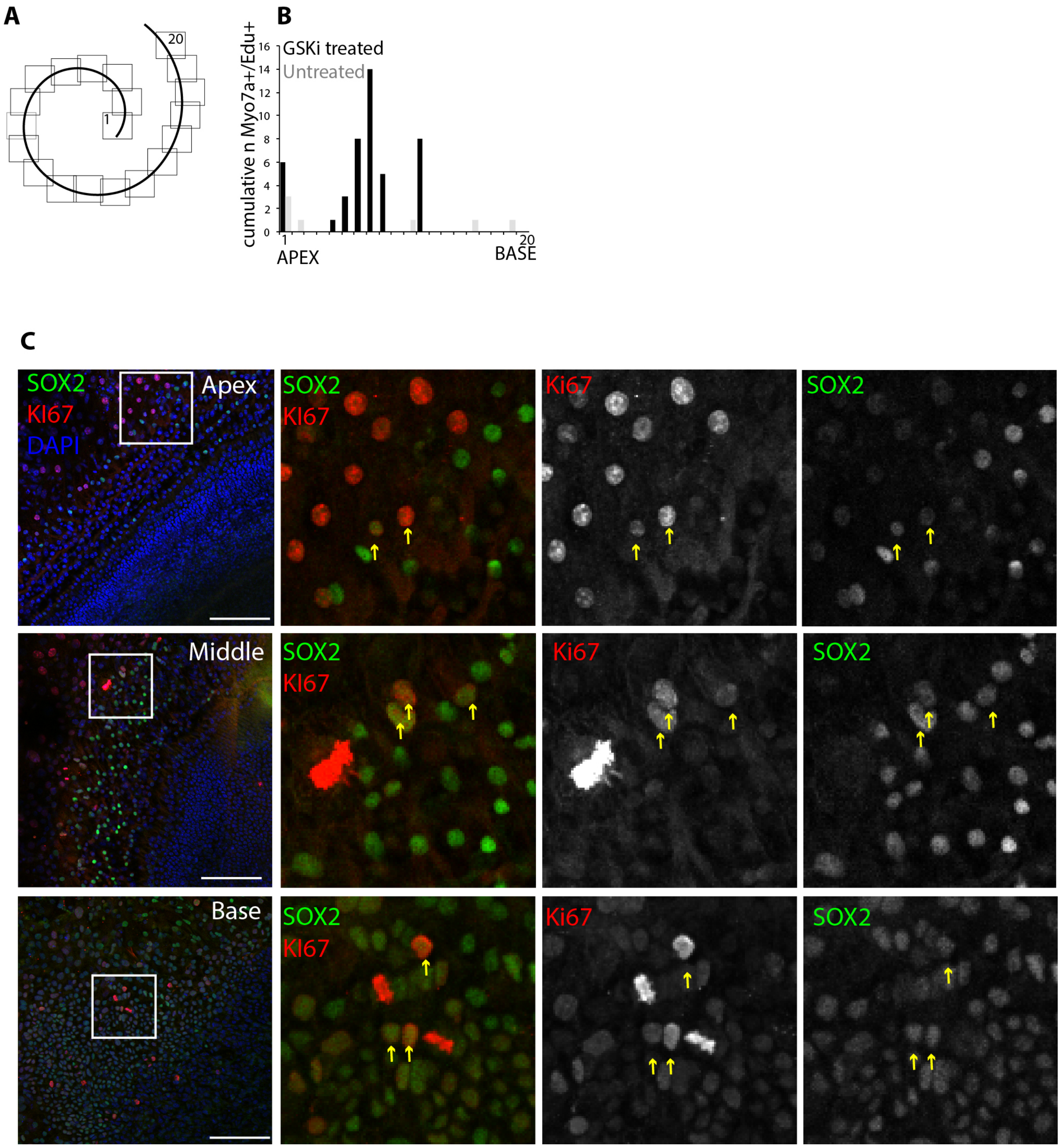

Supplementary S4 Legend

a) Schematic of the imaging strategy. b) Cumulative number of MyoVIIa+/Edu+ hair cells along the organ of Corti (as in schematic a) from 3 different animals (age p3). c) Representative image of organotypic OC cultures from wt animals (age p5), treated for 5 days with 10μM CHIR99021. Cells are immunostained for sox2 (green), Ki67 (red) and DAPI (blu). Scale bar 100μm. Apex, middle and basal turns are shown. White boxed areas are enlarged: Merged images and single channels are displayed. Yellow arrows point as some selected double positive cells.

Supplementary Figure 5

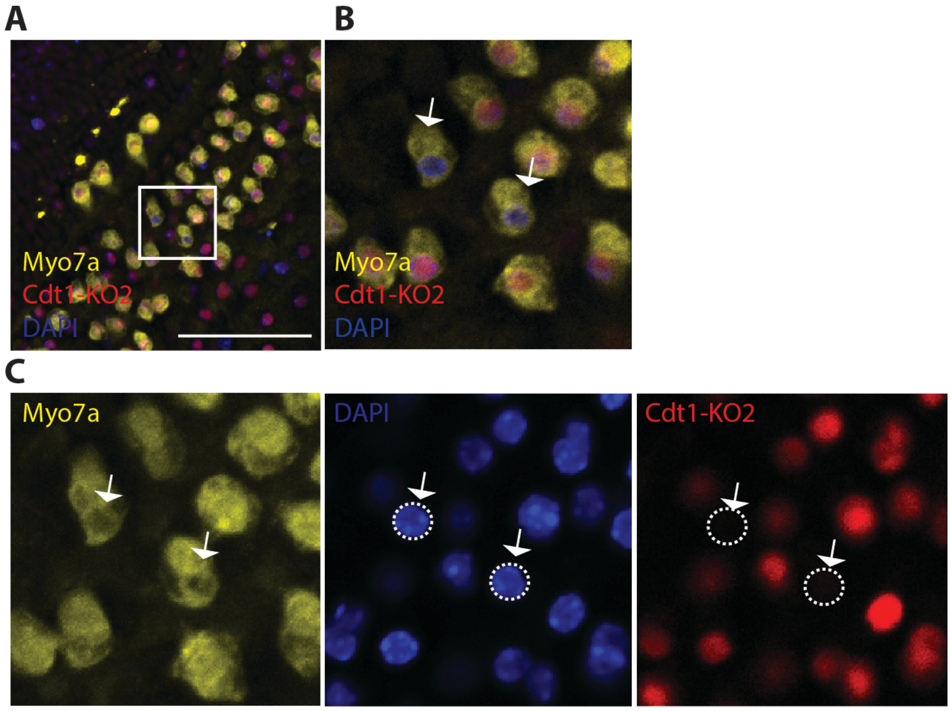

Supplementary S5 Legend

a) Representative image of organotypic OC cultures from Cdt1-KO2 FUCCI animals (age p5) treated for 5 days with 10μM CHIR99021. Immunostaining for MyoVIIa (yellow) is shown. Scale bar 50μm.) Magnification of the white-boxed area in A. c'') single channels from panel b. Arrows and circles indicate MyoVIIa+/Cdt1-KO2- cells.
